# Permeability of the small intestinal mucus for physiologically relevant studies: Impact of mucus location and *ex vivo* treatment

**DOI:** 10.1038/s41598-019-53933-5

**Published:** 2019-11-26

**Authors:** Adam Macierzanka, Alan R. Mackie, Lukasz Krupa

**Affiliations:** 1 Department of Colloid and Lipid Science, Faculty of Chemistry, Gdansk University of Technology, Narutowicza 11/12, 80-233 Gdansk, Poland; 2 Institute of Food Research, Norwich Research Park, Colney Lane, Norwich, NR4 7UA United Kingdom; 3 School of Food Science & Nutrition, University of Leeds, Leeds, LS2 9JT United Kingdom; 4 Department of Gastroenterology and Hepatology with Internal Disease Unit, Teaching Hospital No 1, Chopina 2, 35-055 Rzeszow, Poland; 5 Present Address: Gdansk University of Technology, Gdansk, Poland

**Keywords:** Permeation and transport, Biophysical chemistry, Gastrointestinal models

## Abstract

The small intestinal mucus is a complex colloidal system that coats the intestinal mucosa. It allows passage on nutrients/pharmaceuticals from the gut lumen towards the epithelium, whilst preventing it from direct contact with luminal microorganisms. Mucus collected from intestinal tissue is often used in studies looking at inter-mucosal transport of food particulates, drug carriers, etc. However, detaching the highly hydrated native mucus from the tissue and storing it frozen prior to use may disrupt its physiological microstructure, and thus selective barrier properties. Multiple-particle tracking experiments showed that microstructural organisation of native, jejunal mucus depends on its spatial location in the intestinal mucosa. The inter-villus mucus was less heterogeneous than the mucus covering villi tips in the pig model used. Collecting mucus from tissue and subjecting it to freezing and thawing did not significantly affect (*P* > 0.05) its permeability to model, sub-micron sized particles, and the microviscosity profile of the mucus reflected the overall profiles recorded for the native mucus in the tissue. This implies the method of collecting and storing mucus is a reliable *ex vivo* treatment for the convenient planning and performing of mucus-permeability studies that aim to mimic physiological conditions of the transport of molecules/particles in native mucus.

## Introduction

Mucus is a highly complex viscoelastic medium that provides a defensive barrier for many different mucosal surfaces including the respiratory, reproductive, and gastrointestinal (GI) tracts. The barrier properties of intestinal mucus are complex and vary significantly with location along the GI tract. These variations are associated with the different bacterial loads and the amount of nutrients being absorbed at different locations. The mucus layer comprises two different groups of mucins, secreted and membrane bound. Membrane bound mucins form the glycocalyx that provides an important link between the epithelial cell surface and the secreted gel layer. There has been a significant amount of research on the properties of mucus but much of this work has focussed on the colon^1–3^, with rather less on the properties of mucus from the proximal small intestine^4^. In both cases the primary secreted mucin is MUC2, which has a complex layered structure^5^ where it is tightly adherent^4,6,7^. In the colon, the tightly adherent layer is sufficiently dense to prevent bacteria coming into contact with the epithelium. The biochemical environment and lack of villi in the colon may facilitate the maintenance of this adherent layer. However, in the small intestine this is not the case and the architecture varies longitudinally as the length of the villi decreases from the duodenum to the ilium. The thickness of the mucus layer increases as the villi length decreases. The local variations in structure and concentration of the layer inversely correlate with the thickness as the composition is most heterogeneous in the proximal intestine where the mucus layer is thinner. It is likely that the heterogeneity is caused by the biochemical environment combined with peristaltic motion of the villi generating extensional and compressional flow^8^.

The properties of intestinal mucus have been a topic of research for some time, especially in relation to mucoadhesion^9^. As a result, the structures and properties are quite well described but the heterogeneity of proximal intestinal mucus is still poorly understood. The secreted intestinal mucins are produced by goblet cells and are characterised by their high molecular weight and high proportion of O-linked glycans^10^. Mucus is continuously secreted and as a result, for anything to reach the intestinal epithelium it needs to diffuse through the gradual flow of mucus away from the gut wall or at least more distally along the gut wall^4^. Intestinal secreted mucin is predominantly MUC2, but again there are low concentrations of MUC6 and MUC11 in the small intestine and MUC5B, MUC11, and MUC12 in the large intestine^11^.

The permeability of intestinal mucus is a function of local concentrations of mucin and other large polymers such as DNA and the interactions between these polymers and the material moving through it^12^. The pore size of intestinal mucus has been thought to be of the order of 100 nm^5^. However, measurements using particle tracking have shown that interactions between the mucus and the diffusing particles is at least as important as the pore size in defining permeability^13–15^. These articles have shown that significant negative charge on the diffusing particles is important to avoid adhesion to the mucus, and that this charge can be imparted by the absorption of bile salts. It was also shown that the viscosity was generated by entanglement causing the chains to begin reptating. This was in broad agreement with a model of polyelectrolyte combs with entangled backbones and side-chains. It was also shown that other types of cross-liking agents such as polyphenols could cause gastric mucin to gel, but this was not shown with MUC2.

Mucus collected from the surface of porcine mucosal tissue is often used in studies simulating interactions with ingested foods/pharmaceuticals or intestinal microbiota in the human gut as the physiology of the porcine GI tract is considered most similar to humans^16,17^. Although this might be a convenient way of obtaining sufficient amounts of mucus for various experimental set-ups, concerns have been raised about physiological relevance of studies that use mucus removed from tissue^18^. This is due to the risk of possible, significant alteration of mucus microstructure and microrheology, caused by intense shearing that may occur during the detaching from tissue, or a phase separation (water loss) that may appear in the mucus during storage involving freezing.

In this article, we have investigated the heterogeneity of small intestinal mucus *in situ ex vivo*, using particle tracking. The aim was to show the extent of variations in the native mucus microstructure caused by the spatial location of the measurements in the complex topography of the mucosal tissue. Another goal was to investigate the effect of removal of mucus from the intestinal tissue and freezing/thawing on mucus permeability in order to assess the applicability of the collection/storage method in preserving physiological characteristics of the native mucus regarding barrier properties or inter-mucosal transport.

## Results

### Impact of native mucus location in the intestinal tissue on particulate transport

Experiments on gastrointestinal mucus that has been detached from tissue are useful for evaluating the influence of luminal compounds (e.g. type of drug and/or drug carrier^19–21^, type of dietary fibre^22,23^, etc.) or the source of mucus (e.g. gastric, small intestinal or colonic^12,24,25^) on colloidal transport through the mucus. However, they usually do not take into account possible variations in transport characteristics between different locations in native mucus overlaying the complex topography of the mucosal epithelium, which may be caused by physiological differences in local microstructure of the mucus^4,12^. This may limit the physiological relevance of studies solely using detached mucus.

In this work, we investigated how the structure of the mucus enveloping the small intestinal mucosa can impact on its permeability to sub-micron sized particles such as partially digested food particles or colloidal delivery systems, and their transport. Fluorescent, 500-nm latex beads were used as model particles in the experiments simulating the passage of particles from the lumen of the small intestine^14,15^. The probe particles were incubated with 11 mM BS before being used in multiple-particle tracking experiments on the mucus. This was in order to mimic the physiological concentration of BS in the postprandial, small intestinal lumen of adult humans^26^. The incubation with BS caused a substantial increase in negative charge of the particles, from ca. −20 mV in the absence of BS to ca. −50 mV with BS (Table 1), which suggests the BS adsorbed to the surface of the beads.

**Table 1:** The ζ-potential of dispersions of particles in the presence or absence of 11 mM bile salts (BS). Data are presented as means ± SD.

Dispersed particles	BS	ζ-potential (mV)
500-nm latex beads	−	−19.1 ± 2.0
500-nm latex beads	+	−49.2 ± 2.2
Dispersed fresh *ex vivo* mucus	−	−11.7 ± 0.9
Dispersed fresh *ex vivo* mucus	+	−12.5 ± 1.3
Dispersed stored *ex vivo* mucus	−	−11.6 ± 0.8
Dispersed stored *ex vivo* mucus	+	−12.9 ± 0.9

**Figure 1:**
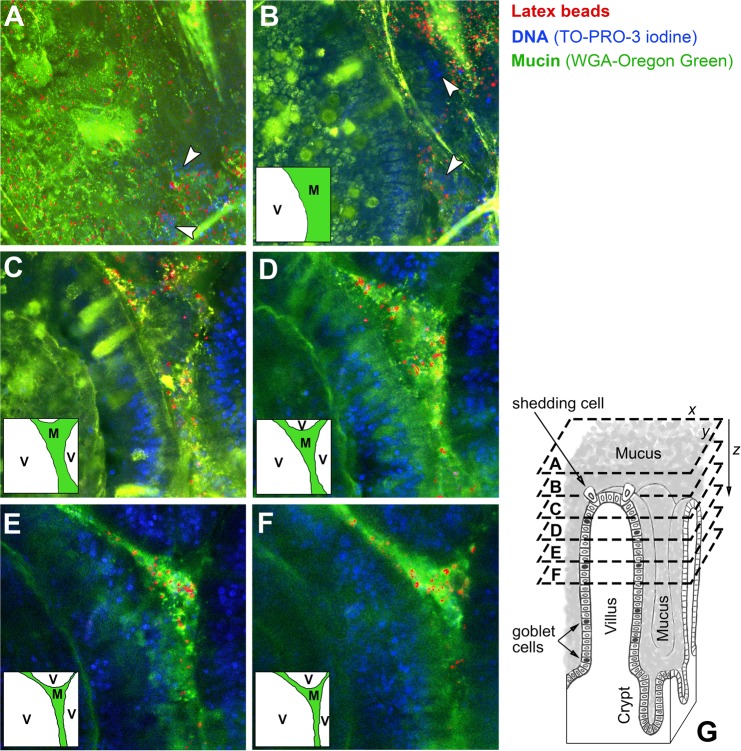
Latex beads in the native mucus covering the porcine small intestinal mucosal tissue. Representative confocal microscopy showing localisation of 500-nm latex beads (red), pre-incubated with 11 mM BS, in the jejunal mucosal tissue visualised at different z-positions along the villi tips–intestinal crypts axis. The specimen was stained for mucin with WGA-Oregon Green (green) and with TO-PRO-3 iodine for DNA (blue). Image A (200 × 200 μm) shows the surface mucus covering the tissue (i.e. the mucus located 5–10 μm below the top of the mucus surface, and above the villi tips). The following images (200 × 200 μm) show x-y scans of the specimen at increasing z-distances from the surface of the mucus: (**B**) 20 μm, (**C**) 40 μm, (**D**) 60 μm, (**E**) 80 μm and (**F**) 100 μm below the top of the mucus surface (as schematically depicted in (**G**)). The epithelial surface of the villus tip is shown in (**B**). As the scanning was acquired further down towards the intestinal crypts, cross-sections of villi and the mucus filling the inter-villus space were clearly seen (**C**–**F**). The insets in (**B**–**F**) are guide for x-y locations of villi (‘V’) and mucus (‘M’). Arrows in (**A**) and (**B**) indicate DNA of the epithelial cells that have been shed from tips of villi into the mucus, whereas the blue channel in (**C**–**F**) shows DNA of cells in villi.

**Figure 2:**
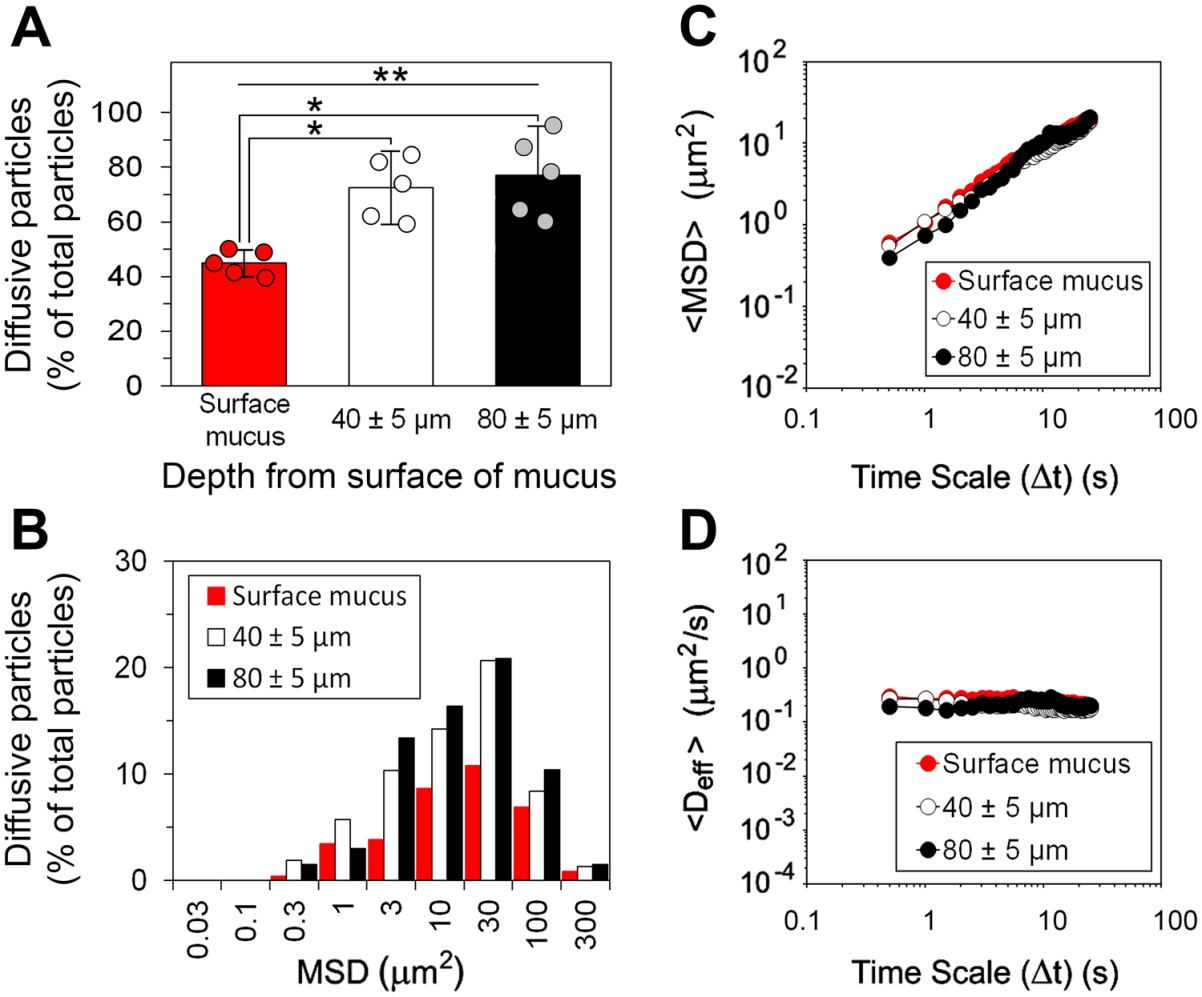
Transport rates and distribution of 500-nm latex beads (pre-incubated with 11 mM BS) in the native mucus covering porcine small intestinal (jejunal) mucosal tissue. Impact of the mucus spatial location in the tissue: (i) the surface mucus layer, and the mucus located (ii) 40 ± 5 μm and (iii) 80 ± 5 μm below the top of the surface mucus (see Fig. 1G for schematic representation). (**A**) Proportions of diffusive beads (data shown as means ± SD). (**B**) Distributions of mean-square displacement (MSD) values obtained for individual diffusive beads at the time scale Δt = 25 s. (**C**) Ensemble mean-square displacements (<MSD>) for the diffusive fractions of beads as a function of Δt, and (**D**) ensemble diffusivities (<D_eff_>) for the diffusive fractions of beads as a function of Δt. *N* = 5, with >100 beads per experiment (*P < 0.05, by Student’s *t*-test; **P < 0.05, by two-way ANOVA). All measurements were done at 37 ± 0.1 °C.

In the next step, the dispersion of latex beads was gently introduced onto the surface of mucosal tissue. We used sections of freshly excised proximal jejunum, with an intact, native mucus layer attached to the mucosal epithelium to look at the permeability of the surface mucus layer that covers tips of villi (i.e. the mucus that is directly exposed to luminal contents under physiological conditions of the gut) as well as the mucus located below that surface and between villi. Figure 1 shows representative confocal micrographs of the native mucus in fresh, unfixed jejunal tissue that was incubated with 500-nm latex beads as described in the experimental section. The particles were evenly distributed in the surface mucus (Fig. 1A) but only 44.9 ± 4.9% of their total number was found diffusing in multiple-particle tracking experiments (Fig. 2A). Motion of the remaining particles in that location was restricted by the mucus matrix over the time scale of experiment. Some latex beads were able to penetrate deeper into the inter-villus spaces (Fig. 1B–F). Those have been tracked in x-y planes at the z-positions of 40 ± 5 µm and 80 ± 5 µm below the surface of mucus. The proportions of diffusive particles in those regions were significantly (*P* < 0.05, by Student’s *t*-test) higher than in the surface mucus: 72.5 ± 13.5% and 77.0 ± 17.9%, respectively, of the total numbers of particles observed there (Fig. 2A). This suggests differences in microstructural organisation and/or composition of mucus between those locations and the surface region. Statistical analysis of the results from all three locations of the native mucus by ANOVA confirmed significant (*P* < 0.05) difference between the groups. Similarly as observed recently^12^, the mucus located above the tips of villi (Fig. 1A,B) was found to contain extracellular DNA that most likely derived from apoptotic enterocytes, which had been shed into the surrounding mucus from the villi tips. The distances travelled by individual diffusing particles varied widely (Fig. 2B). Nevertheless, the ensemble mean-square displacement data (<MSD>, Fig. 2C) showed that the average distance increased proportionally with time over the time scale analysed. The MSD data obtained from trajectories of individual beads have been further converted to diffusion coefficients (effective diffusivities, D_eff_), and finally ensemble diffusion coefficients (<D_eff_>) calculated for families of diffusive particles in the three locations analysed (Fig. 2D). The <D_eff_> values were constant over time (0.16 ± 0.02 μm^2^ s^−1^ for the surface mucus, and 0.10 ± 0.03 μm^2^ s^−1^ and 0.11 ± 0.02 μm^2^ s^−1^ for the inter-villus mucus located 40 ± 5 µm and 80 ± 5 µm below the surface of mucus, respectively), indicating that the mobile particles expressed free diffusion.

### Effect of *ex vivo* mucus treatment on particulate transport

Having examined variations in permeability of the native mucus overlaying the mucosal tissue, we looked at how detaching the mucus from freshly excised tissue might affect the transport of particles diffusing in it. We also tested whether freezing, storing and thawing of the collected mucus can impact on its microstructure and permeability to particles. As for the experiments on the native mucus, the probe 500-nm latex beads were pre-treated with BS before incubating with the *ex vivo* mucus for multiple-particle tracking experiments. The treatment caused an increase in their negative charge as mention earlier (Table 1). In contrast, the BS had no considerable effect on the net charge of the *ex vivo* fresh and stored mucus, with the ζ-potential ranging from −11 mV to −13 mV before and after exposing the mucus to the BS (Table 1). This suggests there was very limited interaction of the bio-surfactants with the mucus. This is consistent with the previous result observed for a lower concentration of BS^14^.

**Figure 3:**
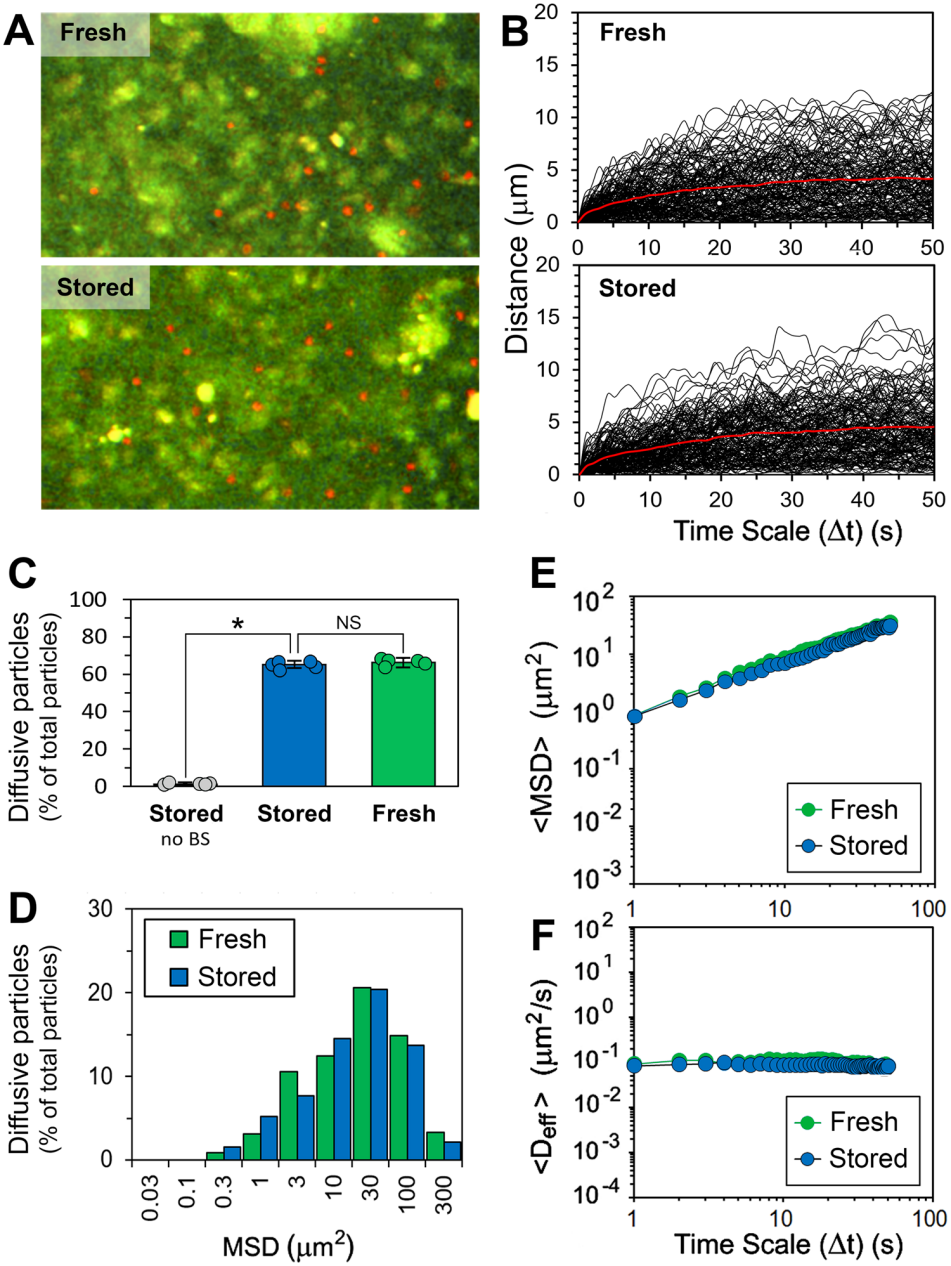
Transport rates and distribution of 500-nm latex beads (pre-incubated with 11 mM BS) in the porcine *ex vivo* fresh and stored, small intestinal (jejunal) mucus. (**A**) Representative confocal micrographs (160 × 90 µm) of the fresh and stored mucus specimens stained for mucin with WGA-Oregon green (green channel), with BS-treated latex beads (red channel) distributed within the mucus. (**B**) Variations in distance travelled by individual beads diffusing in the mucus over the time scale (Δt) of experiment. Red lines show average distance. (**C**) Proportions of diffusive beads in the fresh and stored mucus (data shown as means ± SD). Data obtained for beads that were not pre-incubated with BS is also shown. (**D**) Distributions of mean-square displacement (MSD) values obtained for individual diffusive beads at the time scale Δt = 50 s. (**E**) Ensemble mean-square displacements (<MSD>) for diffusive fractions of beads as a function of Δt, and (**F**) ensemble diffusivities (<D_eff_>) for diffusive fractions of beads as a function of Δt (*n* = 5, with 100–150 beads per experiment). Student’s *t*-test: NS, not significant (*P* > 0.05); *P < 0.001. All measurements were done at 37 ± 0.1 °C.

The latex particles were incubated with mucus as explained in the experimental section, and subsequently analysed by the multiple-particle tracking method. Representative micrographs of WGA-stained mucus specimens with latex beads distributed within the mucus are shown in Fig. 3A. Time series recorded for beads diffusing in the *ex vivo* mucus are also presented (Fig. 3B), and show variations in distance travelled by individual beads over the time scale of experiment. In order to better illustrate the motion of beads within mucus, a representative video clip of the confocal time-lapse microscopy has been included in this report as Supplementary Video S1. The video (125 × 100 µm frame) shows transport of BS-treated 500-nm latex beads in the stored *ex vivo* mucus at 37 ± 0.1 °C over the course of 200 s, displayed at 15× speed. The pre-incubation of the probe particles with BS, and the resulting increase in their negative charge, had a profound effect on their diffusion in the stored *ex vivo* mucus. This too was consistent with the previous studies^14^, and the high negative charge imparted by BS to the particles was assumed to substantially increase the electrostatic repulsion between them and the mucus network, and hence largely prevent mucoadhesion of particles. Here we determined proportions of diffusive particles. Their number increased to 65.2 ± 1.9% of the total number of particles in the presence of BS, from only 0.9 ± 0.6% found for the control conditions (Fig. 3C), where the particles had substantially reduced ζ-potential in the absence of BS (Table 1). Thus for the latter, most of particles (ca. 99%) were found immobilised by the mucus structure over the time scale of experiment. More importantly, there was no influence of the freezing and thawing of the mucus on the overall number of particles capable of diffusing in it. In the freshly collected and examined mucus, the diffusive population of 66.1 ± 2.5% latex beads was recorded (Fig. 3C), which was not significantly (*P* > 0.05) different from the number found for the mucus that had been stored frozen before examination. This suggests the storage conditions had no effect on mucus microstructure with regard to penetrability to the probe particles. In order to investigate it further, we examined trajectories of individual particles. As shown in Fig. 3D, the distances travelled by individual diffusive beads varied largely after the 50-s time scale examined, with MSD values ranging from 0.3 µm^2^ to 300 µm^2^. However, this pattern was consistent for both, the fresh mucus and the stored mucus. The ensemble MSD (<MSD>) values for individual populations of particles in the function of time are given in Fig. 3E, and show that the freezing/thawing had almost no effect on the average distance travelled by the beads in mucus. In the next step, <D_eff_> values were calculated from the MSD data. The diffusive particles showed free diffusion, expressed by constant <D_eff_> values in time (Fig. 3F). The values were very similar between the fresh mucus and the stored mucus (0.09 ± 0.02 μm^2^ s^−1^ and 0.09 ± 0.01 μm^2^ s^−1^, respectively).

### Microviscosity of mucus

**Figure 4:**
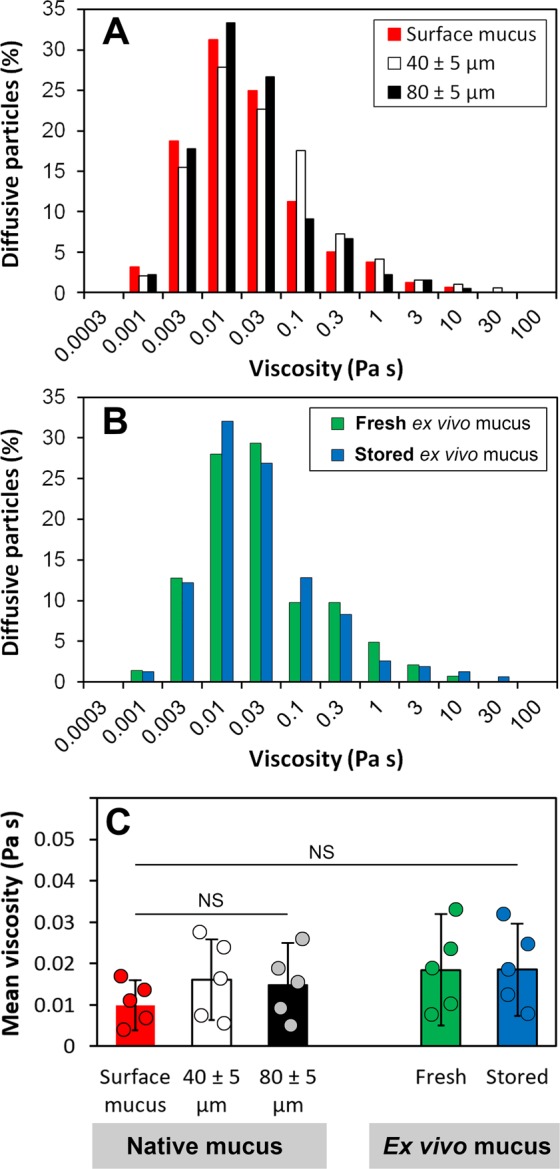
The Stokes-Einstein microviscosity of porcine native and *ex vivo* small intestinal (jejunal) mucus. (**A**) Distribution of the apparent viscosity of the native mucus as determined from diffusion of 500-nm latex beads (diffusive fractions; see Fig. 2A) in various spatial locations of the mucosal tissue; i.e. in the surface mucus layer, as well as in the mucus located 40 ± 5 μm and 80 ± 5 μm below the top of the surface mucus (see Fig. 1G for schematic representation). (**B**) Distribution of the apparent viscosity for the fresh and stored *ex vivo* mucus as determined from diffusion of 500-nm latex beads (diffusive fractions; see Fig. 3C). (**C**) Comparison of the mean viscosity values (±SD) for the native and *ex vivo* mucus. *N* = 5, with 70–110 beads per experiment (NS, not significant (*P* > 0.05), by two-way ANOVA).

The D_eff_ data obtained for particles diffusing in different locations of the native mucus overlaying mucosal tissue (Fig. 2) was used to derive the microviscosity of mucus in those locations. The viscosity experienced by individual particles has been calculated using the Stokes–Einstein equation, and its distributions shown in Fig. 4A. The diffusing particles revealed very broad distribution of viscosity, ranging from 1 mPas to 10–30 Pas. The calculations of mean viscosity values returned 9.9 ± 6.1 mPas for the surface mucus, and 16.1 ± 9.7 mPas and 14.9 ± 10.1 mPas for the mucus located 40 ± 5 µm and 80 ± 5 µm below the mucus surface, respectively (Fig. 4C). The mean values were not significantly different (*P* > 0.05).

The D_eff_ data obtained from particles expressing free diffusion was also used to examine microviscosity of the *ex vivo* fresh and stored mucus samples in regions were diffusion could be detected. As in the native mucus, the diffusing latex beads showed broad distributions of viscosity (Fig. 4B). The mean viscosity values were found to be very similar between the fresh mucus and the stored mucus (18–19 mPas, Fig. 4C). They did not differ significantly (*P* > 0.05) from the microviscosity of the native mucus samples.

## Discussion

Small intestinal mucus comprises a vast range of molecular compounds and amongst them are two major biopolymers, mucin glycoproteins (mainly MUC2^1,11^) and extracellular DNA^12^, which are large enough to contribute to the viscoelastic properties of the mucus. The mucins are actively secreted by the goblet cells located along villi^27^, whereas the extracellular DNA can appear in the mucus mainly as a result of epithelial cell turnover. Enterocyte cells are continuously shed from the villi tips^28,29^, and their nuclei progressively degrade and ‘unfold’ in the mucus. Hence, the extracellular DNA can be embedded predominantly in the mucus adjacent to the villi tips, which is directly exposed to the intestinal lumen. This is especially true for the DNA that is still in the form of only partially-fragmented nuclei (i.e. before they get degraded further to smaller fragments)^12^, which therefore can act as a volume restricted to diffusion of sub-micron sized particles in the surface mucus. The above suggest that the particulate transport characteristics may differ between various spatial locations of mucus in the tissue depending on local microstructure, which is driven by a complex topography of the mucosal tissue.

Variations in functional organisation of mucus overlaying different parts of the mouse GI tract have been studied by Ermund *et al*.^30^. Colon and stomach were shown to have an outer (exposed to the lumen) mucus layer and an inner mucus layer that was adherent to the epithelium, but only in the stomach, the inner layer was fully penetrable to particles the size of bacteria (0.5–2-µm beads were used). The small intestinal mucus was shown as a single layer that was penetrable to the particles. However, spatial organisation of the mucus was not investigated, and the study focused on qualitative, rather than quantitative, illustration of mucus penetrability. In our previous report^12^, we showed that extracellular DNA can substantially contribute to restricting diffusion of microparticles in *ex vivo* scraped, jejunal mucus from adult pigs and 2-week old piglets. Multiple-particle tracking experiments revealed that diffusion of probe particles (500-nm beads) was considerably enhanced after treating the *ex vivo* mucus samples with DNase. The fraction of diffusive beads increased in the pig mucus from 0.6% to 64% and in the piglet mucus from ca. 30% to 77% after the treatment. The initial difference in permeability was partially attributed to the higher DNA content in the pig mucus compared to the piglet mucus. The above suggest that extracellular DNA can substantially contribute to the barrier properties of the intestinal mucus layer. Here, we show that BS-coated, 500-nm latex beads can penetrate the native mucus overlaying the epithelium of freshly excised porcine (adult pig) small intestinal tissue. More importantly, this is the first time the spacial location of native mucus has been found to play an important role in mucus permeability. Thus, motion of particles was restricted to a larger extent in the surface mucus covering the tips of villi than in the mucus filling inter-villus spaces (Fig. 2A). This might be attributed to the extracellular DNA fragments localised mainly in the surface mucus where they could contribute to the local structure/composition of the mucus, as explained above. Accumulation of extracellular DNA in the surface mucus adjacent to the tips of small intestinal villi was illustrated in our previous report^12^.

Our results give a quantitative illustration of the model of continuous mucus coverage in the small intestine^4^. In the model, the loosely-adherent mucus overlaying the villi tips and exposed to the intestinal lumen was proposed to be largely disrupted by a peristaltic motion. The disruption was shown to result in very high degree of heterogeneity of the loosely-adherent mucus, with formation of areas of agglomerated mucin network. This, coupled with the aforementioned accumulation of partially degraded DNA from shed epithelial cells in the loosely-adherent, surface mucus layer^12^, may explain why we could only observe free diffusion for ca. 45% of particles in the surface mucus (Fig. 2A). The remaining 55% of particles were immobilised most likely in areas of aggregated mucin/DNA network. In the mucus coverage model^4^, only the mucus network closest to the epithelium was suggested to remain undisturbed (tightly-adherent layer). In the present work, we could confirm existence of such a network of mucus in areas between villi, where significantly (*P* < 0.05) fewer probe particles were found to be immobilised by the mucus matrix (Fig. 2A). All the above suggest a lower degree of heterogeneity of the inter-villus mucus than the surface mucus. However, the variations in heterogeneity detected from the transport of 500-nm latex beads might only be relevant for motion of relatively large particulates in the mucus (e.g. (sub)micron-sized particles of partially digested food, bacteria). Despite the observed differences in proportions of diffusive/immobilised beads, those particles that were able to diffuse experienced a similar viscosity in both, the surface mucus and the inter-villus mucus locations. The comparison of mean microviscosity values did not show a significant difference (*P* > 0.05, Fig. 4C). This suggests similar microrheological properties of areas available for diffusion in different mucus locations. This also suggests that local differences in heterogeneity of mucus network might be of less importance when considering transport of smaller objects (e.g. nutrient molecules) through the mucus.

As highlighted recently^31^, the advantage of carrying out multiple-particle tracking experiments on mucus that has been collected from the surface of intestinal mucosa is the relative ease of focusing on the particles within a thick mucus gel layer reconstituted in experiment set-up. This is often in contrast to direct particle tracking in mucus produced by cell cultures or in some excised mucosal tissue explants (e.g. small biopsies), where the concern about whether the particles being imaged are within mucus, as opposed to the fluid above or cells below the mucus layer, might arise due to relatively thin or discontinuous layers of mucus attached to the examined cellular structures. However, the intestinal mucus is an example of a highly hydrated gel, and its collection and storage, especially involving freezing, may affect the microstructural and microrheological properties of this hydrocolloid. Freezing followed by thawing can cause substantial syneresis of gels. This is because during freezing, aqueous gels can undergo separation into fractions due to the formation of ice crystals, such that the dispersed phase is concentrated in a non-ice phase. When thawed, the ice crystals melt to form a mixture of water and freeze concentrated gel. This has been shown to cause syneresis of starch gels^32^. However, the microstructural stability of hydrocolloid gels exposed to freezing and thawing seems to be strongly dependent on the water holding capacity of the biopolymers forming the gels. For example, including a high-molecular weight dextran in starch gels was found to significantly reduce the syneresis rate during freeze-thaw cycles due to restricting the mobility of water in gel matrix^33^. A similar improvement in the freeze/thaw stability of complex hydrocolloid gels was observed after incorporating other biopolymers, pectin and gellan gum, and the polysaccharides were concluded to serve as a “dispersed macromolecular medium” for low-molecular weight substances dissolved in the aqueous phase of the gel^34^.

In our study, storing the mucus frozen before examination did not seem to affect its overall microstructure in terms of penetrability to diffusing particles, which might be due to the presence of mucin and DNA. The hydration of nucleic acids is well known to be essential for their conformation, and controls their biological mechanism of action^35,36^. The water molecules are stable around dsDNA even out to about 0.65 nm, which corresponds to three hydration layers^37^. The strength of these aqueous interactions is usually far greater than those for proteins^38^. Similarly, mucin glycoproteins are known for their exceptional water holding capacity, which is crucial in hydrating and lubricating biological surfaces they cover, including the gastrointestinal epithelium^39,40^. The mucin-associated glycans are essential for the formation of such lubricating layers, and deglycosylation of the mucins that had been extracted from pig stomachs was shown to result in a 3.5-fold decrease in hydration and an increase in friction by two orders of magnitude as compared to the native mucins^41^. The microrheological behaviour of mucin gels depends on degree of hydration and thus is particularly relevant to the transport of particles through mucus^42^. In this study, we found that freezing followed by thawing did not cause separating of aqueous phase from the *ex vivo* mucus gel matrix and its penetrability to probe particles was virtually the same as for the freshly collected mucus (Fig. 3). Additionally, the average percentage of particles capable of diffusing in the *ex vivo* fresh or stored mucus, detached from the tissue (Fig. 3C), was in the range of the results observed for the native surface mucus and the native inter-villus mucus that coated the mucosal tissue (Fig. 2A). The Stokes-Einstein microviscosity profiles for both the collected *ex vivo* mucus (fresh and stored; Fig. 4B) and the native mucus in freshly excised tissue (Fig. 4A) were similar, with no significant difference (*P* > 0.05) in mean viscosity values (Fig. 4C). This shows that the collected *ex vivo* mucus can be used as a reliable model system for studying permeability of an overall native mucus overlaying complex topography of the small intestinal mucosal tissue. The results suggest that removing mucus from the tissue and storing it frozen before experiments does not considerably affect the microstructure of mucus with regard to diffusibility of penetrating particles, so the heterogeneity and the resulting microviscosity of the *ex vivo* mucus restricting movement of diffusive particles are representative of those observer for the native mucus coating the mucosal tissue.

## Conclusions

This work contributes to validating the use of collected mucus in experiments simulating inter-mucosal transport under physiological conditions of the gut. By using our simple collection and storage procedure, the overall microstructural and microrheological properties of mucus can be maintained in terms of penetrability to diffusing particles even if the mucus was stored frozen after collecting. Our findings can aid in the convenient planning and performing of mucus-permeability studies that aim to reflect physiological transport of nutrients from the intestinal lumen towards the mucosal epithelium, as well as an inter-mucosal transport of orally-administrated pharmaceuticals in the small intestine.

## Materials and Methods

### Small intestinal *ex vivo* mucus and mucosal tissue

Fresh pig small intestines were obtained from a local abattoir (H. G. Blakes Ltd, Cotessey, Norfolk, UK), from healthy animals (7–10-month old) that were slaughtered for a commercial meat production process, and therefore any ethical requirements that would be specific for this study were not necessary. The authors obtained permission from the abattoir to use these animal parts in the study. Pigs were routinely fasted prior to despatch for slaughter. This largely reduced the amount of digesta residing in the intestines obtained for this study. Immediately after slaughter, the whole small intestine was removed. Only the proximal jejunum (e.g. from the first meter of the small intestine after the duodenum) was used in further procedures. The intestinal lumen was gently flashed with an ice-cold simulated intestinal fluid (SIF; i.e. oxygenated PBS buffer (Sigma-Aldrich, Poole, UK) completed with 25 mM sodium bicarbonate, 1 mM calcium chloride, 10 mM glucose, 0.02% w/v sodium azide and a mix of protease inhibitors (Roche Diagnostics GmbH, Mannheim, Germany; 1 tablet per 50 mL buffer) at pH 7.4) in order to remove any remaining debris, prevent microbial growth, and inhibit proteolytic enzymes.

For experiments on *ex vivo* mucus, the fresh intestinal segments were carefully opened longitudinally and the mucus gently removed from the mucosal surface with a soft-rubber scraper, and within 20 min from slaughter. Special attention was paid during the process to avoid damaging the mucosal tissue surface. Aliquots of ca. 5 mL were transferred to plastic, screw-capped vials and transported on ice to laboratory (this material has been referred to as the ‘fresh *ex vivo* mucus’ throughout the paper). Experiments on the fresh mucus started within no more than 30 min after collecting the mucus. Alternatively, small aliquots of the *ex vivo* mucus were transferred immediately after collection to 0.5-mL eppendorf tubes, frozen in liquid nitrogen and stored at −80 °C for at least two weeks (this has been referred to as the ‘stored *ex vivo* mucus’ throughout the paper). Aliquots of the stored mucus were incubated for 10 min at RT prior to use.

For experiments on the mucosal tissue, 10-cm sections of fresh jejunum (obtained as above) were carefully clipped at both ends, immediately placed in an ice-cold SIF and transported on ice to laboratory in order to limit disturbing of the native, intact mucus overlaying the mucosal surface. Confocal microscopy experiments on the mucosal tissue started within no more than 30 min, and the mucus examined has been referred to as the ‘native mucus’ throughout the paper.

### Materials

Stock dispersion of red-fluorescent carboxylate-modified latex beads (500-nm diameter, 2.5% w/w aqueous dispersion; cat. no. L3280) was supplied by Sigma-Aldrich (Poole, UK). Two bile salts (BS): sodium taurocholate and sodium glycodeoxycholate (Sigma-Aldrich, Poole, UK; cat. no. T4009 and G9910, respectively) were dissolved together in equimolar quantities in SIF (pH 7.4) to give a stock solution with a total BS concentration of 150 mM. The compounds were selected to mimic the properties of BS in human bile, as described previously^43–45^. Prior to experiments, the BS stock was diluted with SIF and the stock dispersion of latex beads was added to the final concentration of 2.5 × 10^−3^% w/w. The final, total concentration of BS was 11 mM, which corresponded to an average, physiological concentration of BS in the postprandial small intestinal lumen of adult human^26^. The diluted dispersion was incubated at RT for 10 min prior to use. For the particle tracking experiments on the mucosal tissue samples, the dispersion was additionally made up with fluorescent stains, to the concentrations given below.

### Specimen preparation for confocal microscopy

For the experiments on fresh or stored *ex vivo* mucus samples, the mucus samples were stained at RT (5 min) for mucins, using wheat germ agglutinin-Oregon Green 488 conjugate (WGA-Oregon Green, Invitrogen W6748; 1 mg/mL in 0.1 M sodium bicarbonate buffer, pH 8.3), and for DNA with TO-PRO-3 Iodine (1 mM solution in DMSO; Invitrogen, Paisley, UK) at the final concentrations of the dyes of 10 µg/mL and 3 µM, respectively, and placed in a 1-mm deep optical cell. The cell was filled to ca. 80% of its volume with the mucus and carefully covered with a coverslip, so that the coverslip was in contact with the mucus. The remaining volume was gently filled with the dilute aqueous dispersion of the latex beads through an opening in the cell, and the opening finally sealed with a silicon grease. That way, diluting of the mucus gel by the dispersion was prevented, so the boundary between the mucus and the aqueous dispersion was clearly visible and interactions of the beads with the surface and the interior of the mucus phase were able to be monitored, mimicking the passage of particles from the lumen into the mucus layer in the small intestine. If the penetration of beads into the mucus was hindered (in experiments with no BS added to the dispersion of latex beads), the experiment was repeated; the mucus was mixed gently with the diluted dispersion of latex (at high mucus to dispersion ratio, ca. 99: 1 v/v, in order to minimise diluting of mucus), and the optical cell was filled completely with the mixture^12,14^. Specimens were placed on a temperature-controlled microscope stage (37 ± 0.1 °C) and incubated for 20 min prior to particle tracking experiments.

For the experiments on mucosal tissue and the native mucus attached to it, segments of freshly excised proximal jejunum were carefully opened along the mesenteric border. Square-shaped section (ca. 8 × 8 mm) of the tissue was gently removed and immediately placed and immobilised (with villi and mucus facing up) in a laboratory-made square well mounted on a standard microscope slide. Special attention was paid to avoid disturbing the mucus layer during the process and to minimise the time required for the specimen preparation in order to limit exposition to air and possible dehydration. In order to ensure an even distribution of particles over the entire mucus layer, the procedure described by Ensign *et al*.^18^ was used, i.e. a small volume of the diluted latex beads dispersion was carefully placed directly on the mucus surface by suspending droplets of the dispersion from the pipette tip and utilising capillary flow to distribute the dispersion over the mucus layer without disturbing its structure. The dispersion of latex contained BS (11 mM) and fluorescent stains (WGA-Oregon Green at 10 µg/mL, and 3 µM TO-PRO-3 Iodine). The well was gently covered with a coverslip and sealed with silicon grease to prevent sample dehydration. The depth of the well was adjusted, so the coverslip was positioned just above the mucus layer and did not compress the mucosal tissue. Specimen was placed on a temperature-controlled microscope stage (37 ± 0.1 °C) and incubated for 20 min prior to particle tracking experiments.

### Multiple-particle tracking in *ex vivo* mucus and the native mucus covering epithelial surface of the mucosal tissue

Images were captured with a Leica TCS SP confocal laser scanning head mounted on a Leica DMRE upright microscope (Leica Microsystems (UK) Ltd, UK). Fluorescence from the sample was excited at 488 nm and 633 nm. Motion of the beads in the *ex vivo* mucus was recorded at 37 ± 0.1 °C using a 40×/1.25 NA oil-immersion objective, at a temporal resolution of 1 s for 50 s. Specimens were scanned 30–50 μm below the coverslip. The pinhole size was increased to 2 Airy units in order to facilitate the extended tracking of particles in the focal plane. The number of tracked beads was typically kept in the range of 15–30 per field of view in order to avoid their mutual interactions. Trajectories of 100–150 beads per experiment were analysed. Only those beads that managed to enter the mucus matrix during the incubation step were tracked. The particle tracking experiments on the native mucus covering the mucosal tissue were done in a similar fashion but at a temporal resolution of 500 ms for 25 s, and with a 20×/0.5 NA dry objective. In this type of experiment, motion of beads was recorded at various z-positions in the specimen. Typically, the beads found just below the surface of the mucus (i.e. 5–10 μm below the boundary between the aqueous phase, simulating a luminal content, and the surface plane of the mucus covering the tissue) were first examined. Same as for the experiments on the *ex vivo* mucus, only those beads that managed to enter the mucus matrix during the incubation step were tracked. Therefore, the scanning was done 5–10 μm below the top of the mucus surface. This location in a mucosal tissue specimen has been referred to as the ‘surface mucus’ throughout the paper. Subsequently, time-lapse imaging was carried out for x-y focal planes in the mucus located further down from the top (i.e. between the villi and towards the intestinal crypts). Mucus in those locations have been referred to as the ‘inter-villus mucus’. However, the depth of scanning was limited to no more than 100 μm (±5 μm) below the surface of the mucus due to an increase in the laser light scattering and a decline in intensity of the signal recorded for z-distances larger than that threshold. Measurements were repeated for up to 5 different places within a central area of specimen. All the experiments described here were performed at least five times for each condition (i.e., for five individual fresh/stored *ex vivo* mucus specimens or five specimens of jejunal tissue). The measurements were carried out on samples obtained from five individual animals. Each specimen was used for up to 25 min (after the 20-min incubation step). The results are shown as means ± SD and/or distributions of data from measurements, for each condition used. Statistical comparisons between two groups were made using a Student’s *t*-test, and three (or more) groups were evaluated using two-way ANOVA (significance level, *P* < 0.05).

Particle trajectories were analysed by using Image-Pro Analyzer 7.0 software (Media Cybernetics Inc., Silver Spring, MD) and are 2D representations of a 3D transport. Movement of an individual particle centroid was transformed into time-dependent mean-square displacement (MSD), <Δr^2^(Δt)> = <Δx^2^ + Δy^2^>, where Δx and Δy are particle displacements in x and y directions, respectively, and Δt is the time scale over which the displacement was calculated^46–48^. By averaging MSDs from trajectories of many particles with identical Δt, ensemble mean-square displacement <MSD> was calculated for families of particles. Effective diffusivities (diffusion coefficients, D_eff_) were obtained from D_eff_ = MSD/4Δt, and ensemble effective diffusivities <D_eff_> calculated for families of particles by averaging D_eff_ values obtained for individual particles. An individual particle was considered diffusive if its D_eff_ values were constant in time over the time scale of tracking, which meant that its final MSD value was typically ≥0.3 μm^2^. For each condition used in this study, a proportion of diffusive particles (% of total particles) was calculated. For the particles that were undergoing simple diffusion (i.e. the diffusive particles), apparent local microviscosity of mucus (η) was calculated using the Stokes–Einstein equation, D = k_B_T/6πηr, where D is the diffusion coefficient independent of time, k_B_ is the Boltzmann’s constant, T is an absolute temperature in Kelvin and r is the radius of diffusing particle. For each mucus type analysed in this study, the mean microviscosity was calculated by averaging viscosity values obtained for individual particles diffusing in a given type of mucus.

### ζ-potential measurements

The ζ-potential of dispersions of particles (i.e. dispersed mucus, latex beads) was obtained from dynamic light scattering measurements at 37 °C, using the method described before^12^. Prior to analysis, *ex vivo* mucus samples were gently dispersed in SIF (pH 7.4) containing 11 mM BS to give the mucus concentration of ca. 0.1% w/v, whereas the latex dispersion was diluted to the concentration of ca. 1.5 × 10^−3^% w/v. Control samples (i.e. with no BS added) were also analysed. Each sample was analysed at least 20 times and the results displayed as a mean. The results are shown as means ± SD from at least three individual dispersions prepared under the same conditions (dispersions of mucus were obtained by using samples from five individual animals, which were measured separately).

## Supplementary information

Supplementary Information

## Data Availability

All relevant data are within the paper.
